# BGN May be a Potential Prognostic Biomarker and Associated With Immune Cell Enrichment of Gastric Cancer

**DOI:** 10.3389/fgene.2022.765569

**Published:** 2022-01-26

**Authors:** Shiyu Zhang, Huiying Yang, Xuelian Xiang, Li Liu, Huali Huang, Guodu Tang

**Affiliations:** ^1^ Department of Gastroenterology, The First Affiliated Hospital of Guangxi Medical University, Nanning, China

**Keywords:** biomarker, prognostic index, bioinformatics analysis, gastric cancer, immune infiltration

## Abstract

**Background:** Biglycan (BGN) plays a role in the occurrence and progression of several malignant tumors, though its role in gastric cancer (GC) remains unclear. The objective of this study was to investigate BGN expression, its role in GC prognosis, and immune infiltration.

**Material and Methods:** Gene expression data and corresponding clinical information were downloaded from TCGA and GTEx, respectively. We compared the expression of BGN in GC and normal tissues and verified the differential expression via Real-Time PCR and immunohistochemistry. BGN-related differentially expressed genes (DEGs) were identified. Additionally, the relationships between BGN gene expression and clinicopathological variables and survival in patients with GC were also investigated through univariate and multivariate Cox regression analyses. Finally, we established a predictive model that could well predict the probability of 1-, 3-, and 5-years survival in GC.

**Results:** We found a significantly higher expression of BGN in GC than that in normal tissues (*p* < 0.001), which was verified by Real-Time PCR (*p* < 0.01) and immunohistochemistry (*p* < 0.001). The 492 identified DEGs were primarily enriched in pathways related to tumor genesis and metastasis, including extracellular matrix (ECM)-receptor interaction, focal adhesion pathway, Wnt signaling, and signaling by VEGF. BGN expression was positively correlated with the enrichment of the NK cells (r = 0.620, *p* < 0.001) and macrophages (r = 0.550, *p* < 0.001), but negatively correlated with the enrichment of Th17 cells (r = 0.250, *p* < 0.001). BGN expression was also significantly correlated with histologic grade (GI&G2 *vs.* G3, *p* < 0.001), histologic type (Diffuse type *vs.* Tubular type, *p* < 0.001), histologic stage (stage I *vs.* stage II and stage I *vs.* stage III, *p* < 0.001), T stage (T1 *vs.* T2, T1 *vs.* T3, and T1 *vs.* T4, *p* < 0.001) and *Helicobacter pylori* (HP) infection (yes *vs.* no, *p* < 0.05) in GC. High BGN expression showed significant association with poor overall survival (OS) in GC patients (HR = 1.53 (1.09–2.14), *p* = 0.013). The constructed nomogram can well predict the 1-, 3-, and 5-years overall survival probability of GC patients (C-index = 0.728).

**Conclusion:** BGN plays an important role in the occurrence and progression of GC and is a potential biomarker for the diagnosis and treatment of GC.

## Introduction

Gastric cancer (GC) is considered to be the fifth most common malignancy and the third leading cause of cancer-related deaths (Chen et al., 2016; Bray et al., 2018) worldwide. Disappointingly, most patients with stomach cancer are diagnosed with advanced cancer because they lack specific symptoms (Van Cutsem et al., 2016). Because of the poor prognosis of patients with advanced GC, it is imperative to develop new strategies to improve the survival rate of this disease.

Expression of BGN (Biglycan), the gene as proteoglycan-I, was first detected in bone tissue (Gallagher, 1989). BGN is a member of the small leucine-rich proteoglycans (SLPRs) gene family and encodes a protein core that is modified to form a glycoprotein (Chen et al., 2020). BGN is a key component of the ECM; it participates in scaffolding the collagen fibrils and mediates cell signaling (Appunni et al., 2021). Existing studies have demonstrated the role of BGN in tumor proliferation, adhesion and invasion (Cooper and Giancotti, 2019; Hisamatsu et al., 2020; Moreno-Layseca et al., 2019; Yousefi et al., 2021). BGN could induce the epithelial-mesenchymal transition (EMT) of diverse malignancies and is necessary and sufficient to mediate the pro-EMT effect in pancreatic ductal adenocarcinoma (Thakur et al., 2016). BGN is regulated by the transforming growth factor-beta (TGFB) signaling pathway, a key regulator of the EMT process (Yang et al., 2021). Moreover, BGN is believed to enhance the ability of endometrial cancer cells to migrate and invade tissue (Sun et al., 2016) and is also considered a potential EMT biomarker of colorectal cancer (Li et al., 2017). Existing research findings strongly suggest an important role of BGN in the development of tumors. Immunotherapy of tumors has been one of the hot topics in recent years. Several studies have documented significant effects of immunotherapy on tumors (Zhang et al., 2015; Marrelli et al., 2016; Shitara et al., 2019); however, there is no report on immunotherapy of BGN in GC. Moreover, the role of BGN in the prognosis of GC and how BGN affects the immune infiltration of GC remain poorly understood.

In this study, we analyzed the difference in BGN expression between GC and normal patients in the online database by bioinformatics analysis. Thereafter, differentially expressed genes (DEGs) associated with BGN were identified. DEG-related functional enrichment analysis, Gene Set Enrichment Analysis (GSEA) analysis, and immune infiltration analysis were also carried out. We also explored the relationship between BGN gene expression and clinicopathological variables and survival in patients with GC. Finally, a predictive model that could well predict the probability of 1-, 3-, and 5-years survival in GC was established.

## Materials and Methods

### Data Sources

Gene expression data and corresponding clinical information for GC, which included 375 tumor tissues and 32 normal tissues, were downloaded from The Cancer Genome Atlas (TCGA) database (https://portal.gdc.cancer.gov/). Table 1, Table 2 shows the characteristics of patients with GC from the TCGA database. The gene expression of 174 normal tissues was downloaded from GTEx through UCSC XENA (http://xena.ucsc.edu). Fragments Per kilobase per Million (FPKM) RNAseq data were converted into transcripts Per Million reads (TPM), and log2 translated for subsequent analysis. All tissue samples with incomplete clinical data were excluded.

**TABLE 1:** The clinical characteristic of Gastric Cancer.

Characteristic	Levels	Overall
N		375
Gender, n (%)	Female	134 (35.7%)
	Male	241 (64.3%)
Age, n (%)	≤ 65	164 (44.2%)
	>65	207 (55.8%)
T stage, n (%)	T1	19 (5.2%)
	T2	80 (21.8%)
	T3	168 (45.8%)
	T4	100 (27.2%)
N stage, n (%)	N0	111 (31.1%)
	N1	97 (27.2%)
	N2	75 (21%)
	N3	74 (20.7%)
M stage, n (%)	M0	330 (93%)
	M1	25 (7%)
Histological type, n (%)	Diffuse Type	63 (16.8%)
	Mucinous Type	19 (5.1%)
	Not Otherwise Specified	207 (55.3%)
	Papillary Type	5 (1.3%)
	Signet Ring Type	11 (2.9%)
	Tubular Type	69 (18.4%)
Pathologic stage, n (%)	Stage I	53 (15.1%)
	Stage II	111 (31.5%)
	Stage III	150 (42.6%)
	Stage IV	38 (10.8%)
Histologic grade, n (%)	G1	10 (2.7%)
	G2	137 (37.4%)
	G3	219 (59.8%)
Residual tumor, n (%)	R0	298 (90.6%)
	R1	15 (4.6%)
	R2	16 (4.9%)
Primary therapy outcome, n (%)	PD	65 (20.5%)
	SD	17 (5.4%)
	PR	4 (1.3%)
	CR	231 (72.9%)
*H pylori* infection, n (%)	No	145 (89%)
	Yes	18 (11%)
Barretts esophagus, n (%)	No	193 (92.8%)
	Yes	15 (7.2%)
Anatomic neoplasm subdivision, n (%)	Antrum/Distal	138 (38.2%)
	Cardia/Proximal	48 (13.3%)
	Fundus/Body	130 (36%)
	Gastroesophageal Junction	41 (11.4%)
	Other	4 (1.1%)
Age, median (IQR)		67 (58, 73)

R0, No visible or microscopic tumor residue; R1, No visible, but microscopic residual tumor; R2, Visible tumor residue; CR, Complete response; PR, Partial response; SD, Stable disease; PD, Progressive disease.

**TABLE 2:** BGN expression levels in 33 cancers and normal tissues.

Cancers	Groups	Cases (n)	Median	Mean	SD	SE	W value	*p* value
ACC	Normal	128	7.343	7.26	0.923	0.082	8172	**< 0.001**
	Tumor	77	6.139	5.99	1.3	0.148		
BLCA	Normal	28	6.128	6.15	0.876	0.166	4289	**0.029**
	Tumor	407	6.801	6.781	1.66	0.082		
BRCA	Normal	292	6.396	6.357	0.944	0.055	26339.5	**< 0.001**
	Tumor	1099	8.537	8.397	1.084	0.033		
CESC	Normal	13	8.134	7.765	1.141	0.317	3047	**0.001**
	Tumor	306	6.439	6.421	1.524	0.087		
CHOL	Normal	9	7.301	7.344	0.525	0.175	77	**0.015**
	Tumor	36	8.033	8.093	0.922	0.154		
COAD	Normal	349	4.953	5.008	1.43	0.077	23468.5	**< 0.001**
	Tumor	290	6.663	6.57	1.586	0.093		
DLBC	Normal	444	0.692	0.937	0.918	0.044	120	**< 0.001**
	Tumor	47	6.668	6.373	1.394	0.203		
ESCA	Normal	666	5.449	5.469	1.17	0.045	16232	**< 0.001**
	Tumor	182	7.309	7.478	1.416	0.105		
GBM	Normal	1157	4.252	4.213	0.894	0.026	2708	**< 0.001**
	Tumor	166	7.288	7.238	1.027	0.08		
HNSC	Normal	44	5.226	5.365	1.466	0.221	3849.5	**< 0.001**
	Tumor	520	7.499	7.392	1.489	0.065		
KICH	Normal	53	7.619	7.31	1.633	0.224	3143	**< 0.001**
	Tumor	66	5.115	5.301	1.139	0.14		
KIRC	Normal	100	7.668	7.58	1.351	0.135	13892.5	**< 0.001**
	Tumor	531	8.799	8.589	1.356	0.059		
KIRP	Normal	60	7.518	7.339	1.477	0.191	11940.5	**< 0.001**
	Tumor	289	6.349	6.42	1.728	0.102		
LAML	Normal	70	0.604	0.714	0.542	0.065	5826.5	0.646
	Tumor	173	0.731	0.942	0.969	0.074		
LGG	Normal	1152	4.249	4.208	0.891	0.026	136652	**< 0.001**
	Tumor	523	5.114	5.367	1.222	0.053		
LIHC	Normal	160	7.068	7.082	0.787	0.062	43486	**< 0.001**
	Tumor	371	5.844	5.797	1.742	0.09		
LUAD	Normal	347	8.61	8.551	0.992	0.053	118430	**< 0.001**
	Tumor	515	8.079	7.978	1.087	0.048		
LUSC	Normal	338	8.673	8.626	0.954	0.052	124402	**< 0.001**
	Tumor	498	7.754	7.646	1.283	0.058		
MESO	Tumor	87	9.419	9.347	1.451	0.156	−	−
OV	Normal	88	5.938	5.973	1.227	0.131	10400.5	**< 0.001**
	Tumor	427	7.063	7.046	1.443	0.07		
PAAD	Normal	171	4.535	4.645	1.365	0.104	961.5	**< 0.001**
	Tumor	179	9.262	8.96	1.173	0.088		
PCPG	Normal	3	7.449	7.465	0.276	0.159	336	0.497
	Tumor	182	7.195	7.231	1.162	0.086		
PRAD	Normal	152	6.951	6.88	1.146	0.093	40731.5	0.133
	Tumor	496	6.777	6.785	1.027	0.046		
READ	Normal	318	5.075	5.096	1.434	0.08	6420.5	**< 0.001**
	Tumor	93	6.717	6.745	1.51	0.157		
SARC	Normal	2	6.951	6.951	0.004	0.003	−	−
	Tumor	262	9.046	8.692	1.898	0.117		
SKCM	Normal	813	6.711	6.804	1.121	0.039	178330.5	0.054
	Tumor	469	6.905	6.939	1.355	0.063		
STAD	Normal	206	4.383	4.58	1.398	0.096	5987	**< 0.001**
	Tumor	375	7.664	7.601	1.368	0.067		
TGCT	Normal	165	6.33	6.431	0.736	0.057	10324	**0.004**
	Tumor	154	6.82	6.913	1.708	0.138		
THCA	Normal	338	7.84	7.682	1.003	0.055	126239.5	**< 0.001**
	Tumor	512	6.909	6.839	1.116	0.049		
THYM	Normal	446	0.696	0.959	0.975	0.046	698.5	**< 0.001**
	Tumor	119	6.381	6.193	1.806	0.166		
UCEC	Normal	101	7.483	7.393	0.979	0.097	13701	**< 0.001**
	Tumor	181	6.162	6.195	1.531	0.114		
UCS	Normal	78	7.556	7.563	0.821	0.093	1690	**0.018**
	Tumor	57	8.198	7.999	1.436	0.19		
UVM	Tumor	79	6.54	6.415	1.115	0.125	−	−

Bold indicates statistically significant, that is, a *p* value less than 0.05.

### BGN Differential Expression in Pan-Cancer and GC Tissues

We downloaded TPM RNAseq data for tumor tissues (TCGA) and normal tissues (TCGA and GTEx) from the UCSC XENA. The differential expression between tumor and normal tissues was tested by Wilcoxon Rank Sum Test and visualized through boxplots and scatter plots. We also used Receiver Operating Characteristic (ROC) curve to determine the diagnostic value of BGN gene expression for GC.

### Real-Time PCR of BGN Expressions in GC and Adjacent Tissues

Tumor and para-cancer biopsy tissues were collected from 12 consecutive patients that were diagnosed with GC for the first time from the Endoscopy Center of the First Affiliated Hospital of Guangxi Medical University. The body tissues were immediately immersed in RNA protection solution and rapidly stored in a refrigerator at −80°C. No patient was diagnosed with any other malignancy, nor had they received any treatment for the tumor.

### RNA Extraction and Quantitative Real-Time PCR (qRT-PCR)

Total RNA of tissues was extracted using Trizol reagent (R0016, Beyotime Biotechnology Co., Ltd., Shanghai, China, according to the manufacturer’s instructions. Complementary DNAs(cDNAs) were generated from 1 µg RNA PrimeScript™ RT Reagent Kit with gDNA Eraser (RR047A, Takara Bio, Inc.). RT-PCR was conducted via the FastStart Universal SYBR Green Master (ROX) (Roche) in the Applied Biosystems QuantStudio TM Real-PCR System (Q6). Human BGN primers were utilized, and the relative mRNA expression was determined using the comparative Ct method with Glyceraldehyde 3-phosphate dehydrogenase (GAPDH) as the reference gene. The primer sequences were as follows:

BGN-forward: 5′-TGA​CTG​GCA​TCC​CCA​AAG​AC-3′

BGN-reverse: 5′-GAG​TAG​CGA​AGC​AGG​TCC​TC-3′

GAPDH-forward: 5′-GTC​AGC​CGC​ATC​TTC​TTT-3′

GAPDH-reverse: 5′-CGC​CCA​ATA​CGA​CCA​AAT-3′

### Immunohistochemistry

From January 2018 to September 2020, the tumors and adjacent tissues of 80 consecutive patients with GC after surgery in Suqian First People’s Hospital were collected. Patients who had received radiation or chemotherapy prior to surgery and had other malignancies were excluded from the study. After dewaxing, hydration, and thermal repair, the primary antibody against BGN (ab209234, Abcam, 1:2000) was incubated overnight at 4°C followed by incubation with detection polymer for 40 min at room temperature. 3,3′-Diaminobenzidine DAB (P0202, Beyotime Biotechnology co.) was used for signal detection. The images taken under the microscope were analyzed using the IHC profiler plugin of ImageJ software (Varghese et al., 2014). Finally, SPSS version 23.0 software was used to statistic the results.

### Identification of DEGs Between High and Low Expression Groups of BGN

According to the mean value of BGN expression, the data from the TCGA cohort were divided into high expression group and low expression group, and the DESeq2 package (Love et al., 2014) was used for differential analysis. DEGs were defined as having a p.adj <0.05 and |logFC| >1.5. The details of the DEGs were visualized using the volcano map.

### Functional Enrichment Analysis of DEGs

After ID conversion of identified DEGs via or.Hs.eg.db package, further functional enrichment analysis was performed through clusterProfiler package (Yu et al., 2012). Enrichments that satisfied the following conditions were considered significant: p.adj<0.05, and q-value<0.2. DEGs results were employed for gene-set enrichment analyses (GSEA) and building gene-set enrichment plots against the Molecular Signatures Database (MSigDB) hallmark gene sets through the R package, clusterProfiler, and significance was set as an adjusted *p* < 0.05 and FDR<0.25.

### Immune Infiltration

After converting the level 3 HTSe1-FPKM format RNAseq data from the stomach adenocarcinoma (STAD) project of TCGA to TPM format, log2 conversion was performed. After normal tissue samples were removed, data from a total of 375 STAD samples were retained for subsequent analysis. The relative tumor infiltration levels of immune cell types were quantified using ssGSEA of clusterProfilerpackage (Yu et al., 2012) to quantify the relative tumor infiltration levels of immune cell types, and the marker genes of immune cell types for single-sample gene-set enrichment analysis (ssGSEA) were obtained from published signature gene lists (Bindea et al., 2013). Spearman’s Correlation Test was adopted to determine a correlation between BGN and the immune infiltration levels and the association of immune infiltration with the different expression groups of BGN.

### Clinical Correlation Analysis of BGN in Patients With GC

For TCGA data, Wilcoxon signed Rank-Sum test and logistic regression analyses were used to evaluate the relationship between BGN expression and clinicopathological variables. Moreover, univariate and multivariate Cox regression analyses were used to compare the effects of BGN expression and other clinicopathological variables on the overall survival of GC patients. Multivariate Cox regression analysis was used to examine the independent factors affecting the prognosis of GC.

Furthermore, we collected clinicopathological data from 80 patients who underwent immunohistochemistry to evaluate the relationship between BGN expression and clinicopathological variables. Chi-square tests were used to evaluate the relationship between gender, pathological type, residual tumor status, and BGN expression. Fisher’s exact tests were used to evaluate the relationship between pathologic stage, T stage, N stage, primary treatment outcome, and BGN expression. Wilcoxon signed Rank-Sum test was used to evaluate the relationship between age and BGN expression.

### Construction and Verification of Nomogram

The identified independent factors associated with GC prognosis were used to construct a nomogram that predicted the probability of 1-, 3-, and 5-years survival in patients with GC. The prognostic data were obtained from a study by Jianfang Liu(Liu et al., 2018). Nomogram was constructed by R package with the survival and rms package. The Harrell’s concordance index (C-index) was used to quantify the predictive accuracy, which ranges from 0.5 (no predictive power) to 1 (perfect prediction). Furthermore, calibration plots were generated to examine the performance characteristics of the predictive nomogram.

## Results

### BGN Differential Expression in Pan-Cancer and GC Tissues

**FIGURE 1:**
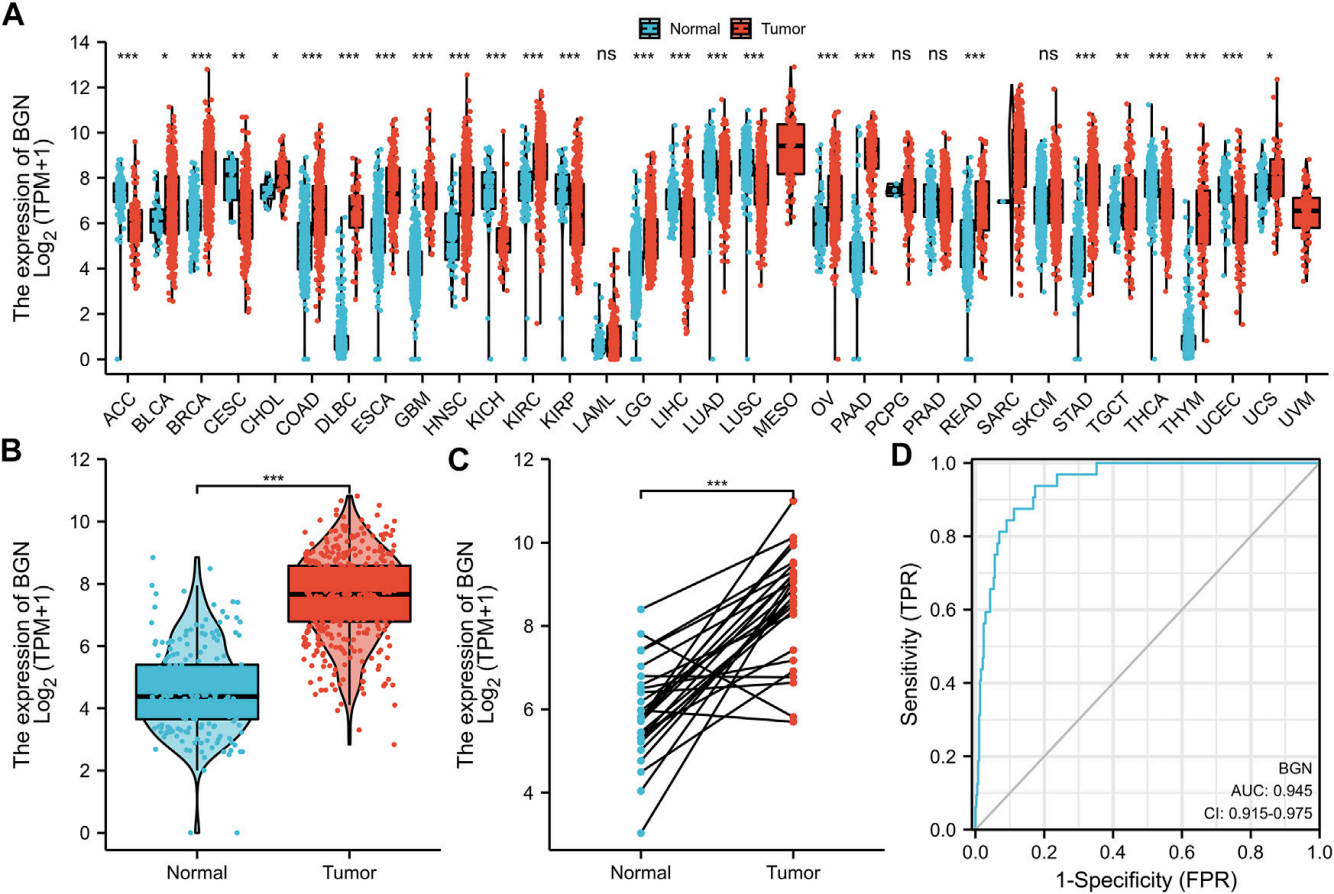
Differential expression of BGN in different tumors and BGN-related differentially expressed genes (DEGs). **(A)** Differential expression of BGN of different cancers compared with normal tissues in the TCGA and GTEx database. **(B,C)** Differential expression of BGN in STAD. **(D)** ROC curve was used to calculate the diagnostic predictive value of BGN expression between STAD and normal tissues. Significance marker: ns, *p* ≥ 0.05; *, *p* < 0.05; **, *p* < 0.01; ***, *p* < 0.001. The abbreviations for 33 cancers are as follows: Adrenocortical carcinoma (ACC); Bladder Urothelial Carcinoma (BLCA); Breast invasive carcinoma (BRCA); Cervical squamous cell carcinoma and endocervical adenocarcinoma (CESC); Cholangiocarcinoma (CHOL); Colon adenocarcinoma (COAD); Lymphoid Neoplasm Diffuse Large B-cell Lymphoma (DLBC); Esophageal carcinoma (ESCA); Glioblastoma multiforme (GBM); Head and Neck squamous cell carcinoma (HNSC); Kidney Chromophobe (KICH); Kidney renal clear cell carcinoma (KIRC); Kidney renal papillary cell carcinoma (KIRP); Acute Myeloid Leukemia (LAML); Brain Lower Grade Glioma (LGG); Liver hepatocellular carcinoma (LIHC); Lung adenocarcinoma (LUAD); Mesothelioma (MESO); Ovarian serous cystadenocarcinoma (OV); Pancreatic adenocarcinoma (PAAD); Pheochromocytoma and Paraganglioma (PCPG); Prostate adenocarcinoma (PRAD); Rectum adenocarcinoma (READ); Sarcoma (SARC); Skin Cutaneous Melanoma (SKCM); Testicular Germ Cell Tumors (TGCT); Thyroid carcinoma (THCA); Thymoma (THYM); Uterine Corpus Endometrial Carcinoma (UCEC); Uterine Carcinosarcoma (UCS); Uveal Melanoma (UVM).

Significant differential expression of BGN was documented in most of the 33 cancers, including in STAD (Figure 1A). The expression of BGN in GC (375 cases from TCGA) was significantly higher than in normal tissues (32 para-cancer tissues from TCGA and 174 normal tissues from GTEx) (*p* < 0.001) (Figure 1B). Similarly, the comparison of 27 tumor tissues in TCGA with the corresponding para-cancer tissues also showed significant expression of BGN in tumor tissues (Figure 1C).

Furthermore, based on the expression profile of TCGA in tumor and normal tissues, a ROC curve of BGN for the diagnosis of GC was plotted. Figure 1D shows that in the prediction of tumor and normal outcomes, the variable BGN showed high accuracy (AUC = 0.945, CI = 0.915–0.975).

### Real-Time PCR and Immunohistochemistry

**FIGURE 2:**
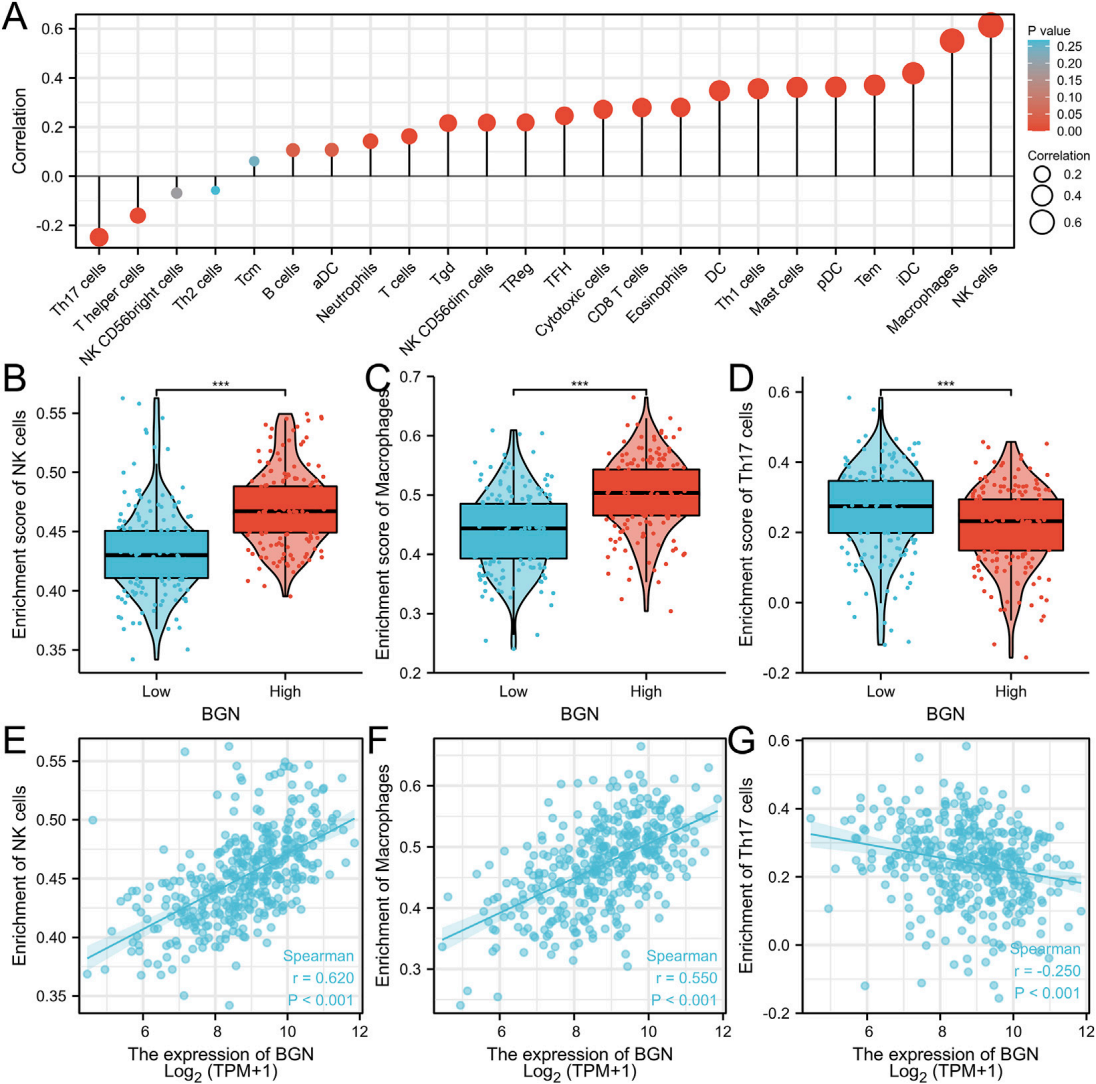
The results of Real-Time PCR and Immunohistochemistry. **(A)** BGN expression in normal tissue (200X). **(B)** BGN expression in gastric cancer tissue (400X). **(C)** BGN expression in normal tissue (200X). **(D)** BGN expression in gastric cancer tissue (400X). **(E)** Relative BGN mRNA level in normal and GC tissues. GC: Gastric cancer. **, *p* < 0.01.

We further verified the BGN expression level using RT-PCR (Figure 2E, *p* = 0.0068) and IHC (Figures 2A–D). The results were consistent with those in the TCGA database, indicating significantly higher levels of BGN expression in GC than that in normal tissues.

### DEGs Identification, Functional Enrichment Analysis and GSEA Analysis of DEGs

**FIGURE 3:**
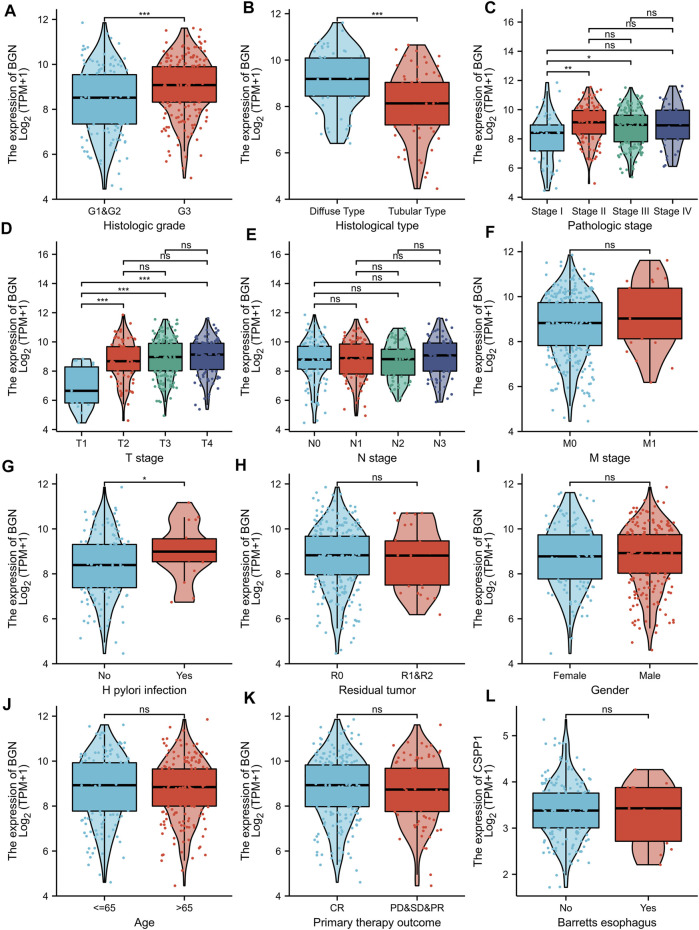
Volcano plot of the DEGs, Functional enrichment analysis and GSEA analysis. **(A)**: **(E)** Volcano plots of the DEGs. Blue represent down-regulated DEGs, red represent up-regulated DEGs. **(B)**: The top three items enriched in biological processes (BP), cellular component (CC), molecular function (MF), and Kyoto Encyclopedia of Genes and Genomes (KEGG) of DEGs. **(C–H)**: Enrichment plots from the gene set enrichment analysis (GSEA). NES, normalized enrichment score; p.adj, adjusted p-value; FDR, false discovery rate.

The volcano map shows the expression of identified DEGs between groups with high and low BGN expression (Figure 3A). of all the 492 DEGs. Of them, 207 were up-regulated, and 285 were down-regulated genes.

In terms of Biological Process (BP), most of the DEGs were enriched in extracellular structure organization, extracellular matrix (ECM) organization, and skin development. In terms of cellular components (CC), DEGs were mostly enriched in the collagen-containing ECM, endoplasmic reticulum lumen, and ECM components. In terms of molecular functions (MF), the DEGs also showed significant association with ECM structural constituent, receptor-ligand activity, and glycosaminoglycan binding. Furthermore, they were found mainly enriched in three KEGG pathways, including protein digestion and absorption, ECM-receptor interaction, focal adhesion pathway (Figure 3B; Table 3). GSEA analysis revealed the following BGN-related enrichment pathways: collagen formulation, immunoregulatory interactions between a lymphoid and a non-lymphoid cell, focal adhesion, ECM glycoproteins, Wnt signaling, and signaling by vascular endothelial growth factor (VEGF), as shown in Figures 3C–H.

**TABLE 3:** GO and KEGG enrichment analysis.

Ontology	ID	Description	Gene ratio	Bg ratio	*p* Value	p.adjust	q value
BP	GO:0043062	extracellular structure organization	51/305	422/18670	1.31e-29	4.07e-26	3.36e-26
BP	GO:0030198	extracellular matrix organization	47/305	368/18670	2.24e-28	3.47e-25	2.86e-25
BP	GO:0043588	skin development	38/305	419/18670	7.95e-18	8.20e-15	6.77e-15
BP	GO:0070268	cornification	20/305	112/18670	1.66e-15	1.28e-12	1.06e-12
BP	GO:0008544	epidermis development	36/305	464/18670	8.33e-15	5.16e-12	4.26e-12
CC	GO:0062023	collagen-containing extracellular matrix	65/318	406/19717	7.31e-46	1.93e-43	1.72e-43
CC	GO:0005788	endoplasmic reticulum lumen	28/318	309/19717	1.66e-13	2.19e-11	1.96e-11
CC	GO:0044420	extracellular matrix component	12/318	51/19717	2.28e-11	2.00e-09	1.79e-09
CC	GO:0005604	basement membrane	14/318	95/19717	3.83e-10	2.53e-08	2.26e-08
CC	GO:0005581	collagen trimer	13/318	87/19717	1.37e-09	7.25e-08	6.47e-08
MF	GO:0005201	extracellular matrix structural constituent	41/290	163/17697	3.73e-37	1.45e-34	1.21e-34
MF	GO:0048018	receptor ligand activity	36/290	482/17697	2.71e-14	5.28e-12	4.41e-12
MF	GO:0005539	glycosaminoglycan binding	22/290	229/17697	2.93e-11	3.79e-09	3.17e-09
MF	GO:0005518	collagen binding	13/290	67/17697	5.41e-11	5.26e-09	4.40e-09
MF	GO:0061134	Peptidase regulator activity	21/290	219/17697	8.66e-11	6.74e-09	5.63e-09
KEGG	hsa04974	Protein digestion and absorption	17/134	103/8076	6.74e-13	1.31e-10	1.17e-10
KEGG	hsa04512	ECM-receptor interaction	10/134	88/8076	1.70e-06	1.66e-04	1.48e-04
KEGG	hsa04510	Focal adhesion	12/134	201/8076	1.20e-04	0.008	0.007
KEGG	hsa00980	Metabolism of xenobiotics by cytochrome P450	7/134	77/8076	2.71e-04	0.013	0.012
KEGG	hsa05204	Chemical carcinogenesis	7/134	82/8076	4.00e-04	0.016	0.014

### Correlation Between BGN Expression and Immune Infiltration

**FIGURE 4:**
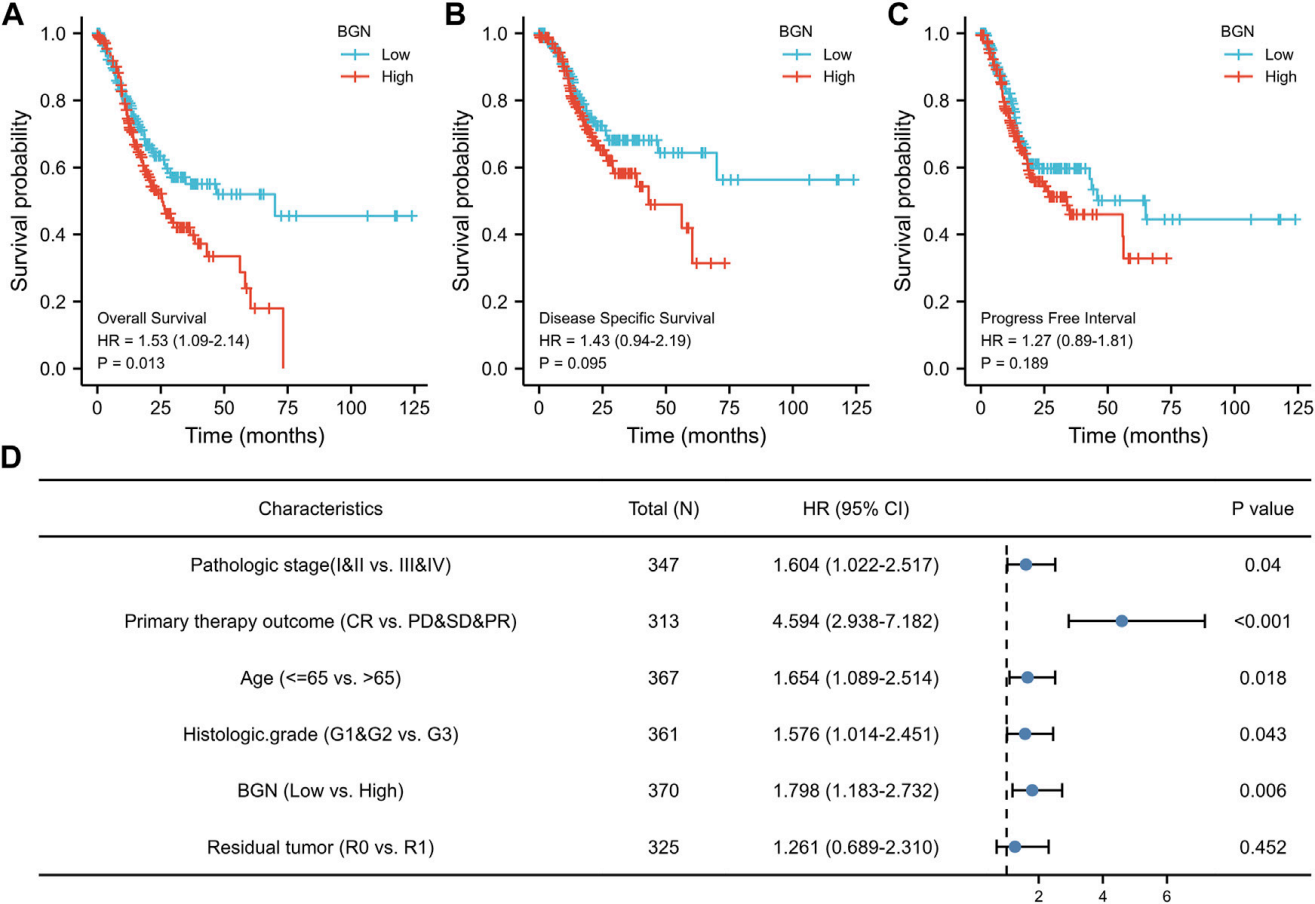
The correlation between BGN expression and immune infiltration. **(A)** Correlation between the relative abundances of immune cells and BGN expression level. The size of dots is positively related to the absolute value of Spearman’s R. **(B-D)** The difference of immune cells (Macrophages, NK cells, and Th17 cells) between the high and low expression groups based on the median value of BGN expression. **(E–G)** The correlation of immune cells (Macrophages, NK cells, and Th17 cells) between the high and low expression groups based on median value of BGN expression.

The BGN expression showed positive correlation with the enrichment of the NK cells (r = 0.620, *p* < 0.001) and macrophages (r = 0.550, *p* < 0.001) but negative correlation with the enrichment of Th17 cells (r = -0.250, *p* < 0.001) (Figures 4A–G; Table 4). The enrichment score of macrophages (High: 0.501 ± 0.061, Low: 0.44 ± 0.066, *p* < 0.001) and NK cells (High: 0.47 ± 0.031, Low: 0.433 ± 0.036, *p* < 0.001) in the group with high BGN expression was significantly higher than that in the group with low BGN expression, while the enrichment score of Th17 cells (High: 0.218 ± 0.111, Low: 0.266 ± 0.12, *p* < 0.001) in the group with high BGN expression was significantly lower than that in the group with low BGN expression (Table 5). The details of immune cell enrichment score in the BGN high expression group and low expression group are shown in Table 5.

**TABLE 4:** Correlation analysis between BGN and immune cells.

Gene	Immune cells	Spearman correlation coefficient	*p* Value
BGN	NK cells	0.620	**<0.001**
BGN	Macrophages	0.550	**<0.001**
BGN	iDC	0.419	**<0.001**
BGN	Tem	0.371	**<0.001**
BGN	pDC	0.363	**<0.001**
BGN	Mast cells	0.362	**<0.001**
BGN	Th1 cells	0.356	**<0.001**
BGN	DC	0.348	**<0.001**
BGN	Eosinophils	0.280	**<0.001**
BGN	CD8 T cells	0.279	**<0.001**
BGN	Cytotoxic cells	0.272	**<0.001**
BGN	Th17 cells	−0.250	**<0.001**
BGN	TFH	0.246	**<0.001**
BGN	TReg	0.219	**<0.001**
BGN	NK CD56dim cells	0.218	**<0.001**
BGN	Tgd	0.216	**<0.001**
BGN	T cells	0.163	**0.002**
BGN	T helper cells	−0.160	**0.002**
BGN	Neutrophils	0.142	**0.006**
BGN	aDC	0.107	**0.038**
BGN	B cells	0.106	**0.040**
BGN	NK CD56bright cells	−0.069	0.185
BGN	Tcm	0.061	0.237
BGN	Th2 cells	−0.057	0.267

Bold indicates statistically significant, that is, a *p* value less than 0.05.

**TABLE 5:** Details of immune cell enrichment score in BGN high expression group and low expression group.

Immune cells	Enrichment scores in high and low expression groups		*p* value
	High (mean ± SD)	Low (mean ± SD)	
Macrophages	0.501 ± 0.061	0.44 ± 0.066	**<0.001**
NK cells	0.47 ± 0.031	0.433 ± 0.036	**<0.001**
Th17 cells	0.218 ± 0.111	0.266 ± 0.12	**<0.001**
aDC	0.394 ± 0.114	0.378 ± 0.119	0.159
B cells	0.231 ± 0.1	0.218 ± 0.112	0.107
CD8 T cells	0.575 ± 0.022	0.564 ± 0.023	**<0.001**
Cytotoxic cells	0.401 ± 0.095	0.36 ± 0.101	**<0.001**
DC	0.36 ± 0.108	0.304 ± 0.102	**<0.001**
Eosinophils	0.391 ± 0.037	0.373 ± 0.039	**<0.001**
iDC	0.433 ± 0.059	0.395 ± 0.054	**<0.001**
Mast cells	0.247 ± 0.087	0.188 ± 0.09	**<0.001**
Neutrophils	0.31 ± 0.092	0.289 ± 0.087	**0.030**
NK CD56bright cells	0.408 ± 0.053	0.412 ± 0.061	0.265
NK CD56dim cells	0.236 ± 0.072	0.208 ± 0.074	**0.001**
pDC	0.544 ± 0.1	0.487 ± 0.103	**<0.001**
T cells	0.392 ± 0.113	0.368 ± 0.114	**0.042**
T helper cells	0.578 ± 0.027	0.587 ± 0.029	**0.004**
Tcm	0.411 ± 0.04	0.406 ± 0.039	0.240
Tem	0.432 ± 0.039	0.406 ± 0.039	**<0.001**
TFH	0.335 ± 0.042	0.316 ± 0.048	**<0.001**
Tgd	0.239 ± 0.041	0.23 ± 0.053	**0.010**
Th1 cells	0.361 ± 0.051	0.328 ± 0.057	**<0.001**
Th2 cells	0.376 ± 0.032	0.375 ± 0.037	0.815
TReg	0.421 ± 0.127	0.376 ± 0.134	**0.002**

Bold indicates statistically significant, that is, a *p* value less than 0.05.

### Relationship Between BGN Expression and Clinicopathological Variables

**FIGURE 5:**
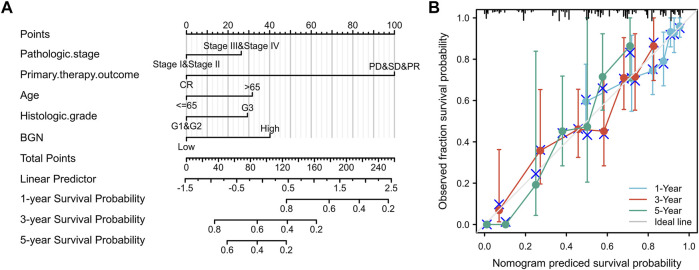
Association with BGN expression and clinicopathological characteristics. **(A)** Histologic grade, **(B)** Histological type, **(C)** Pathologic stage, **(D)** T stage **(E)** N stage, **(F)** M stage, **(G)** *H pylori* infection, **(H)** Residual tumor, **(I)** Gender, **(J)** Age, **(K)** Primary therapy outcome, and **(L)** Barretts esophageal in GC patients in TCGA cohort. TCGA, The Cancer Genome Atlas; GC, gastric cancer.

BGN expression was remarkably correlated with histologic grade (Figure 5A, GI&G2 *vs.* G3, *p* < 0.001), histologic type (Figure 5B, Diffuse type *vs.* Tubular type, *p* < 0.001), histologic stage (Figure 5C, stage I *vs.* stage II and stage I *vs.* stage III, *p* < 0.001), T stage (Figure 5D, T1 *vs.* T2, T1 *vs.* T3, and T1 *vs.* T4, *p* < 0.001) and *Helicobacter pylori* (HP) infection (Figure 5G, yes *vs.* no, *p* < 0.05) in gastric cancer (GC). However, the following clinicopathological features showed no significant association with BGN expression: M stage, N stage, residual tumor, gender, age, primary therapy outcome, and Barrett’s esophagus (Figures 5F,H–L, *p* > 0.05).

The results in Table 6 showed that BGN expression was remarkably correlated with pathologic stage (*p* = 0.008), T stage (*p* = 0.001), histologic type (*p* < 0.001), and histological grade (*p* = 0.025) in 80 GC patients who underwent immunohistochemistry, but was not significantly associated with gender (*p* = 0.802), N stage (*p* = 0.232), residual tumor (*p* = 0.323), primary therapy outcome (*p* = 0.655), anatomic neoplasm subdivision (*p* = 0.905), and age (*p* = 0.600).

**TABLE 6:** The relationship between BGN expression and clinicopathological variables in 80 patients underwent immunohistochemistry.

Characteristic	Low	High	p
n	40	40	0.802
Gender (M/F), n (%)			
F	10 (12.5%)	12 (15%)	
M	30 (37.5%)	28 (35%)	
Pathologic stage, n (%)			**0.008**
I	11 (13.8%)	2 (2.5%)	
II	16 (20%)	14 (17.5%)	
III	13 (16.2%)	24 (30%)	
T stage, n (%)			**0.001**
T1	12 (15%)	1 (1.2%)	
T2	5 (6.2%)	4 (5%)	
T3	23 (28.7%)	32 (40%)	
T4	0 (0%)	3 (3.8%)	
N stage, n (%)			0.232
N0	10 (12.5%)	11 (13.8%)	
N1	12 (15%)	5 (6.2%)	
N2	7 (8.8%)	12 (15%)	
N3	11 (13.8%)	12 (15%)	
Histological type, n (%)			**< 0.001**
Diffuse Type	6 (7.5%)	22 (27.5%)	
Mucinous Type	1 (1.2%)	5 (6.2%)	
Papillary Type	6 (7.5%)	5 (6.2%)	
Signet Ring Type	8 (10%)	6 (7.5%)	
Tubular Type	19 (23.8%)	2 (2.5%)	
Histological grade, n (%)			**0.025**
G1 & G2	24 (30%)	13 (16.2%)	
G3	16 (20%)	27 (33.8%)	
Residual tumor, n (%)			0.323
R0	26 (32.5%)	31 (38.8%)	
R1 & R2	14 (17.5%)	9 (11.2%)	
Primary therapy outcome, n (%)			0.655
CR	27 (33.8%)	32 (40%)	
PD	8 (10%)	5 (6.2%)	
PR	2 (2.5%)	1 (1.2%)	
SD	3 (3.8%)	2 (2.5%)	
Anatomic neoplasm subdivision, n (%)			0.905
Antrum	8 (10%)	8 (10%)	
Cardia	15 (18.8%)	13 (16.2%)	
Fundus/Body	15 (18.8%)	18 (22.5%)	
other	2 (2.5%)	1 (1.2%)	
Age (years), meidan (IQR)	63 (58, 70.5)	66 (58, 71.25)	0.600

R0, No visible or microscopic tumor residue; R1, No visible, but microscopic residual tumor; R2, Visible tumor residue; CR, Complete response; PR, Partial response; SD, Stable disease; PD, Progressive disease.

Bold indicates statistically significant, that is, a *p* value less than 0.05.

### Association With BGN Expression and Prognosis of Patients With GC

**FIGURE 6:**
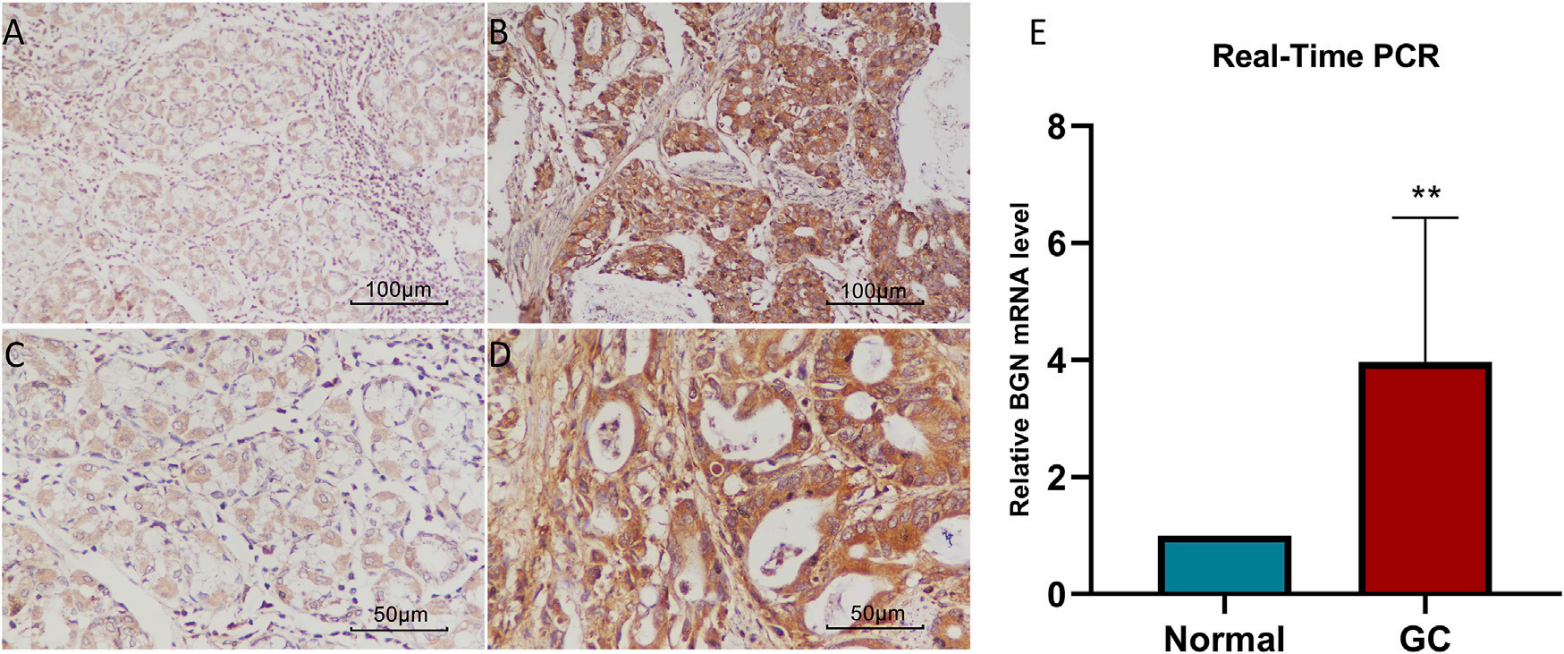
The association between BGN expression and prognosis of patients with Gastric Cancer. **(A)** Overall Survival. **(B)** Disease Specific Survival. **(C)** Progress Free Interval. **(D)** Results of multivariate Cox regression analysis of the relationship between Overall Survival and clinicopathological variables in patients with gastric cancer. HR: Hazard Ratio. CI: Confidence Interval.

The results of survival analysis revealed significant association of greater BGN expression with poor Overall Survival (OS) in GC patients (Figure 6A, HR = 1.53 (1.09–2.14), *p* = 0.013), but no significantly association with Disease Specific Survival (DSS) (Figure 6B, HR = 1.43 (0.94–2.19), *p* = 0.095), and Progress Free Interval (PFI) (Figure 6C, HR = 1.27 (0.89–1.81), *p* = 0.189).

In order to eliminate the influence of other clinicopathological variables on OS of GC, multivariate Cox regression analysis was performed to identify independent factors affecting OS of GC. Table 7 and Figure 6D show that pathologic stage (stage I &II *vs.* stage III &IV, HR (95% CI) = 1.604 (1.022–2.517), *p* = 0.040), primary therapy outcome (CR *vs.* PD &SD &PR, HR (95% CI) = 4.594 (2.938–7.182), *p* < 0.001), age (≤65 *vs.* >65 years, HR (95% CI) = 1.654 (1.089–2.514), *p =* 0.018), histologic grade (G1 & G2 *vs.* G3, HR (95% CI) = 1.576 (1.014–2.451), *p =* 0.043), and BGN (low *vs.* high, HR (95% CI) = 1.798 (1.183–2.732), *p =* 0.006) had significant correlation with OS rates in patients with GC. However, BGN expression showed no association with poor DSS and DSS PFI (Tables 8; Tables 9).

**TABLE 7:** Univariate regression and multivariate survival method (Overall Survival) of prognostic covariates in patients with Gastric Cancer

Characteristics	Total(N)	Univariate analysis		Multivariate analysis	
		Hazard ratio (95% CI)	*p* Value	Hazard ratio (95% CI)	*p* Value
Pathologic.stage	347
Stage I&Stage II	164	Reference			
Stage III&Stage IV	188	1.947 (1.358–2.793)	**<0.001**	1.604 (1.022–2.517)	**0.040**
Primary.therapy.outcome	313
CR	231	Reference			
PD&SD&PR	86	4.228 (2.905–6.152)	**<0.001**	4.594 (2.938–7.182)	**<0.001**
Residual.tumor	325
R0	298	Reference			
R1&R2	31	3.445 (2.160–5.494)	**<0.001**	1.261 (0.689–2.310)	0.452
Age	367
≤ 65	164	Reference			
>65	207	1.620 (1.154–2.276)	**0.005**	1.654 (1.089–2.514)	**0.018**
Histologic.grade	361
G1&G2	147	Reference			
G3	219	1.353 (0.957–1.914)	0.087	1.576 (1.014–2.451)	**0.043**
Gender	370
Female	134	Reference			
Male	241	1.267 (0.891–1.804)	0.188		
Race	320
White	238	Reference			
Asian&Black or African American	85	0.801 (0.515–1.247)	0.326		
BGN	370
Low	188	Reference			
High	187	1.494 (1.070–2.087)	**0.019**	1.798 (1.183–2.732)	**0.006**

R0, No visible or microscopic tumor residue; R1, No visible, but microscopic residual tumor; R2, Visible tumor residue; CR, Complete response; PR, Partial response; SD, Stable disease; PD, Progressive disease.

Bold indicates statistically significant, that is, a *p* value less than 0.05.

**TABLE 8:** Univariate regression and multivariate survival method (Progress Free Interval) of prognostic covariates in patients with Gastric Cancer

Characteristics	Total(N)	Univariate analysis		Multivariate analysis	
		Hazard ratio (95% CI)	*p* Value	Hazard ratio (95% CI)	*p* Value
Pathologic.stage	349
Stage I&Stage II	164	Reference			
Stage III&Stage IV	188	1.676 (1.154–2.435)	**0.007**	1.202 (0.787–1.834)	0.395
Primary.therapy.outcome	315
CR	231	Reference			
PD&SD&PR	86	8.041 (5.465–11.832)	**<0.001**	8.297 (5.319–12.941)	**<0.001**
Residual.tumor	326
R0	298	Reference			
R1&R2	31	3.469 (2.127–5.656)	**<0.001**	1.384 (0.797–2.401)	0.248
Age	369
≤ 65	164	Reference			
>65	207	0.858 (0.603–1.221)	0.395		
Histologic.grade	363
G1&G2	147	Reference			
G3	219	1.540 (1.057–2.245)	**0.025**	1.632 (1.064–2.503)	**0.025**
Gender	372
Female	134	Reference			
Male	241	1.638 (1.099–2.440)	**0.015**	1.404 (0.889–2.217)	0.145
Race	322
White	238	Reference			
Asian&Black or African American	85	1.061 (0.688–1.637)	0.787		
BGN	372
Low	188	Reference			
High	187	1.280 (0.897–1.825)	0.174		

R0, No visible or microscopic tumor residue; R1, No visible, but microscopic residual tumor; R2, Visible tumor residue; CR, Complete response; PR, Partial response; SD, Stable disease; PD, Progressive disease.

Bold indicates statistically significant, that is, a *p* value less than 0.05.

**TABLE 9:** Univariate regression and multivariate survival method (Disease Specific Survival) of prognostic covariates in patients with Gastric Cancer

Characteristics	Total(N)	Univariate analysis		Multivariate analysis	
		Hazard ratio (95% CI)	*p* Value	Hazard ratio (95% CI)	*p* Value
Pathologic.stage	331
Stage I&Stage II	164	Reference			
Stage III&Stage IV	188	2.146 (1.352–3.404)	**0.001**	1.500 (0.874–2.575)	0.141
Primary.therapy.outcome	310
CR	231	Reference			
PD&SD&PR	86	8.697 (5.439–13.908)	**<0.001**	9.129 (5.214–15.984)	**<0.001**
Residual.tumor	314
R0	298	Reference			
R1&R2	31	5.142 (3.014–8.771)	**<0.001**	1.901 (1.022–3.534)	**0.042**
Age	346
≤ 65	164	Reference			
>65	207	1.211 (0.797–1.840)	0.371		
Histologic.grade	340
G1&G2	147	Reference			
G3	219	1.338 (0.862–2.078)	0.194		
Gender	349
Female	134	Reference			
Male	241	1.573 (0.985–2.514)	0.058	1.338 (0.765–2.341)	0.307
Race	305
White	238	Reference			
Asian&Black or African American	85	1.097 (0.656–1.836)	0.724		
BGN	349
Low	188	Reference			
High	187	1.444 (0.945–2.206)	0.089	1.528 (0.931–2.510)	0.094

R0, No visible or microscopic tumor residue; R1, No visible, but microscopic residual tumor; R2, Visible tumor residue; CR, Complete response; PR, Partial response; SD, Stable disease; PD, Progressive disease.

Bold indicates statistically significant, that is, a *p* value less than 0.05.

### Construction and Validation of Nomogram

**FIGURE 7:**
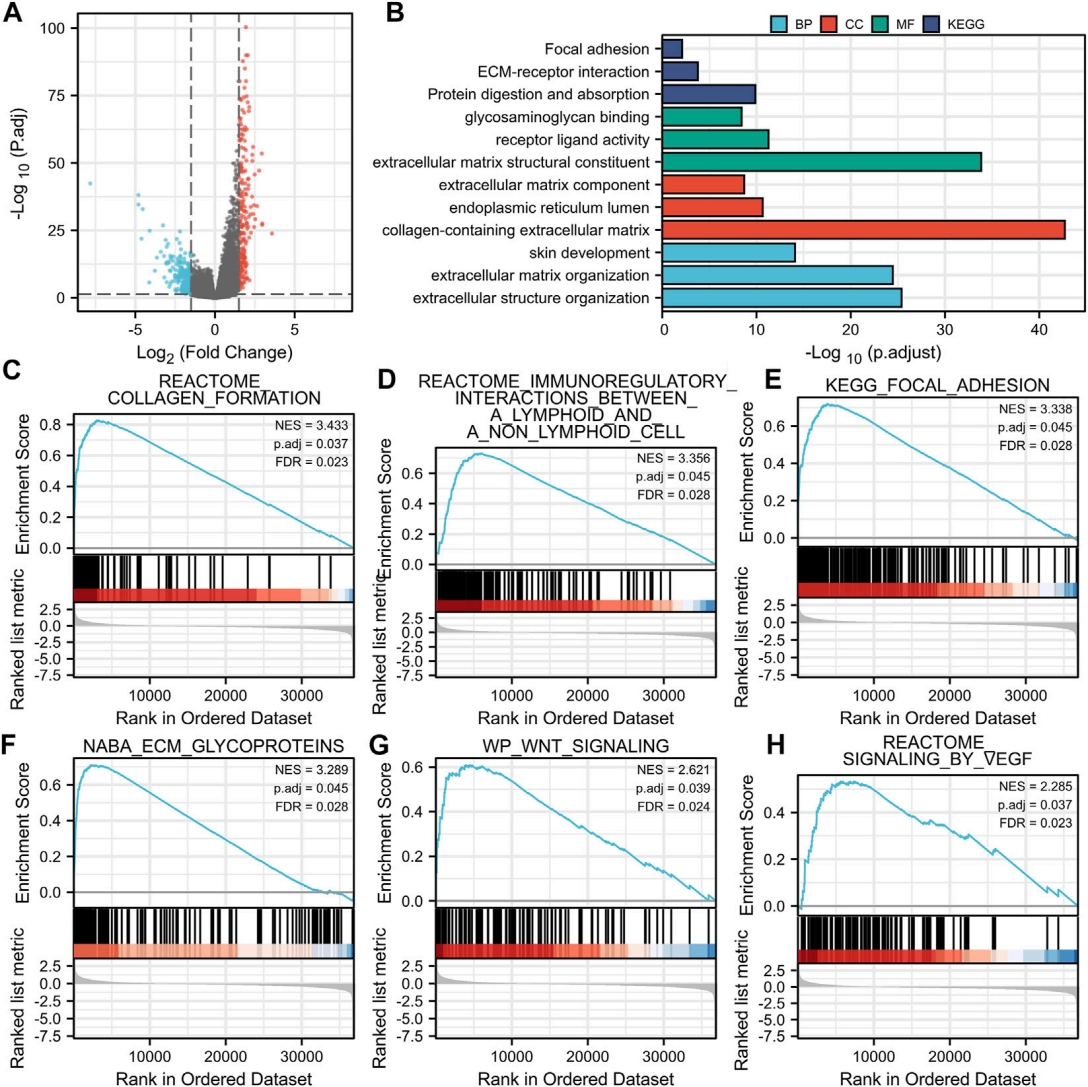
A quantitative method to predict GC patients’ probability of 1-, 3-, and 5-years OS. **(A)** A nomogram for predicting the probability of 1-, 3-, and 5-years OS for GC patients. **(B)** Calibration plots of the nomogram for predicting the probability of OS at 1, 3, and 5 years. GC, gastric cancer; OS, overall survival.

A nomogram to predict 1-, 3-, and 5-years’ OS probability was constructed on the basis of multivariate Cox regression analysis. In it, five variables, namely pathologic stage, primary therapy outcome, age, histologic grade, and BGN expression level, were used. Figure 7A depicts 11 rows in the nomogram, with the rows ranging from 2 to 6 representing the above variables. The points of the five variables were added up to the total points, which were displayed in row 7 and corresponded to the linear predictor in the prediction of 1-, 3-, and 5-years survival probability in row 8. The C-index was used to quantify the predictive accuracy, ranging from 0.5 (no predictive power) to 1 (perfect prediction). The C-index of this nomogram was 0.728 (0.705–0.752), indicating that the prediction was in good agreement with the actual survival probability. The nomogram calibration plot (Figure 7B) also suggests that the nomogram was well-calibrated, with the mean predicted probabilities close to observed probabilities.

## Discussion

In the current study, we compared the expression level of BGN in tumor tissues from TCGA and normal tissues from TCGA and GTEx. The results demonstrated differential expression of BGN in most of the 33 tumors and significant expression in GC tissues. Similar results were obtained on comparison of the GC tissues in TCGA with the matched normal tissues. The expression level of BGN in GC tissues was significantly higher as compared with normal tissues (*p* < 0.001). RT-PCR and IHC also verified this association (*p* < 0.01). The AUC of the ROC curve to predict the diagnostic value of BGN for GC was 0.945 (0.915–0.975), suggesting greater expression of BGN expression in GC diagnosis. The above results suggest that BGN may be a new biomarker for GC.

In addition, 492 BGN-related DEGs, including 207 up-regulated and 285 down-regulated genes, were identified. GO and KEGG enrichment analyses on DEGs were also done. In terms of BP, DEGs were mostly enriched in extracellular structure organization, ECM organization, and skin development. In terms of CC, DEGs were mostly enriched in collagen-containing ECM, endoplasmic reticulum lumen, and ECM components. Also, the DEGs were significantly associated with ECM structural constituent, receptor-ligand activity, and glycosaminoglycan binding in terms of MF. DEGs showed significant enrichment in three KEGG pathways of protein digestion and absorption, ECM-receptor interaction, focal adhesion. ECM plays a key role in the cell microenvironment and in maintaining normal cell activity (Giussani et al., 2019). Recent studies have shown a close correlation of ECM to tumor progression, including in the avoidance of apoptosis, the regulation of cell growth, the promotion of tumor angiogenesis, and the acquisition of invasion and metastasis ability (Pickup et al., 2014; Poltavets et al., 2018; Eble and Niland, 2019). The disorder of collagen, a key component of ECM, correlates with malignant tumor (Levental et al., 2009). Changes in the levels of metabolites related to protein digestion and absorption also have a key role in the development of cancer (Mo et al., 2020). GSEA enrichment analysis revealed that BGN-related DEGs were significantly enriched in collagen formulation (Nissen et al., 2019), immunoregulatory interactions between a lymphoid and a non-lymphoid cell (Sautès-Fridman et al., 2019), focal adhesion (Eke and Cordes, 2015), ECM glycoproteins (Mohan et al., 2020), Wnt signaling (Bugter et al., 2021), and signaling by VEGF (Apte et al., 2019), which were significantly related to the tumor. Considering the above findings, we speculate that BGN-related genes may be involved in the occurrence and progression of GC, and BGN may be a potential therapeutic target for GC.

Immunotherapy of tumors has been one of the hot topics over recent years. The use of Trastuzumab as immunotherapy has been shown to prolong overall survival in patients with HER2-positive GC (Shitara et al., 2019). In several clinical trials (Zhang et al., 2015), adoptive cell therapy has also demonstrated promising results against GC. A high incidence of somatic mutations in GC patients suggests ideal candidacy of Trastuzumab for immunotherapy (Marrelli et al., 2016). These results give us more confidence in the treatment of stomach cancer. However, due to the high complexity of the immune microenvironment of GC, the identification of biomarkers associated with GC require greater attention in the future (Zhao et al., 2019). The BGN expression was positively correlated with the enrichment of the NK cells (r = 0.620, *p* < 0.001) and macrophages (r = 0.550, *p* < 0.001) but was negatively correlated with the enrichment of Th17 cells. This indicates that the improvement of innate immunity is accompanied by the decrease of adaptive immunity. Macrophages, a type of immune cell present in large numbers in most tumor types, play an important regulatory role in promoting the development of malignancy (Noy and Pollard, 2014). Macrophages were recruited by inflammatory signals released by cancer cells in primary and metastatic tumors and differentiated into tumor-associated macrophages (TAMs) that promote tumor progression (Qian et al., 2011; Arwert et al., 2018). A large number of Th17 cell infiltrates were reported in different tumor types, including ovarian cancer (Miyahara et al., 2008), hepatocellular carcinoma (Zhang et al., 2009), colorectal cancer (Tosolini et al., 2011), and multiple myeloma (Prabhala et al., 2010). An abundance of Th17 cells in hepatocellular carcinoma and colorectal cancer showed association with poor prognosis (Kryczek et al., 2009). The results indicate that in the occurrence and development of GC, numerous immune cell infiltration changes occur, which may play a certain regulatory role.

BGN expression showed a significant correlation with histologic grade, histologic type, histologic stage, T stage, and *Helicobacter pylori* (HP) infection in patients with GC. Thus, GC patients with high BGN expression may have poorer histological types, lower tumor differentiation, more advanced tumor development, and may show greater association with HP infection. Furthermore, survival analysis suggested a significant correlation of high BGN expression with poor OS. Multivariate Cox regression analysis was conducted to exclude the influence of other variables. This analysis also showed that pathologic stage, primary therapy outcome, age, histologic grade, and BGN expression level are independent risk factors for OS in GC. These findings strongly suggest the key role of BGN in the development of GC, leading to a poor prognosis of GC.

A nomogram was established to predict 1-, 3-, and 5-years survival probability of GC patients by including the above five independent survivorship risk factors, namely pathologic stage, primary therapy outcome, age, histologic grade, and BGN expression. Our nomogram can predict the OS probability of GC patients very well (C-index = 0.728). The calibration map shows that the nomogram’s predicted OS probability matches the actual probability. Because of the very uncertain prognosis of tumor patients, understanding the risk stratification of patients with tumors correctly (Gratian et al., 2014) becomes crucial. Our nomogram based on independent factors related to the survival of GC patients can predict the OS probability of GC patients and can be widely used in clinical practice Cs-Szabó et al., 1995, Vuillermoz et al., 2004.

## Conclusions and Limitations

Overall, the findings of the current research are summarized below:

First, we reported and verified the differential expression of BGN in GC and normal tissue and concluded that the occurrence, progression, and prognosis of GC were significantly correlated with BGN. Second, BGN is a good biomarker for the proper diagnosis of GC. Third, BGN-related changes in the tumor microenvironment and immune invasion may play an important role in the occurrence and progression of GC. Finally, as our nomogram could predict the survival probability of GC patients, it may be widely used in clinical practice. Due to the limited conditions, we could not study molecular subtypes. This issue will be addressed in future research.

## Data Availability

The datasets presented in this study can be found in online repositories. The names of the repository/repositories and accession number(s) can be found in the article/Supplementary Material.
